# Life and death: the destiny of *Phytophthora sojae* determined by a receptor-like kinase

**DOI:** 10.1007/s44154-023-00132-y

**Published:** 2023-11-21

**Authors:** He Wang, Wen-Ming Wang, Jing Fan

**Affiliations:** State Key Laboratory of Crop Gene Exploration and Utilization in Southwest China, Sichuan Agricultural University, Chengdu, 611130 China

**Keywords:** *Phytophthora sojae*, PsRLK6, Oospore, Microbe-associated molecular patterns, Plant immunity

## Abstract

Leucine-rich repeat receptor-like kinases (LRR-RLKs) are well known to act in plant growth, development, and defense responses. Plant LRR-RLKs locate on cell surface to sense and initiate responsive signals to a variety of extracellular stimuli, such as microbe-associated molecular patterns (MAMPs) released from microorganisms. LRR-RLKs are also present in microbes and function in microbial growth and development, but their roles in communicating with hosts are largely unknown. A recent study published in Nature Communications uncovered that a microbial LRR-RLK, PsRLK6, is required for oospore development in the sexual reproduction of *Phytophthora sojae*, an oomycete pathogen that causes root and stem rot in soybean. Meanwhile, PsRLK6 is recognized as a novel type of MAMP by an unknown plant LRR receptor-like protein and triggers immune responses in soybean, tomato, and *Nicotiana benthamiana*. The findings reveal dual roles of a pathogen LRR-RLK in determining both life through sexual reproduction and death through triggering plant immunity.

## Main text

Microbe-associated molecular patterns (MAMPs) are recognized by pattern recognition receptors (PRRs) in plants, such as leucine-rich repeat receptor-like kinases (LRR-RLKs) and receptor-like proteins (LRR-RLPs), triggering plant immunity. MAMPs are known to be a variety of molecules secreted or released from bacteria, fungi, and oomycetes, including oligosaccharides, peptides, peptidoglycans, oligogalacturonides, and lipids (Yu et al. 2017). For example, two well-known MAMPs, a conserved 22 amino acid epitope of bacterial flagellin and a N-acetylated 18 amino acid peptide of elongation factor Tu (EF-Tu) in bacterial cells, are sensed by the Arabidopsis LRR-RLKs FLS2 and EFR, respectively (Gómez-Gómez & Boller 2000, Kunze et al. 2004). A virulence protein, XEG1, produced by the oomycete pathogen *Phytophthora sojae*, is detected as a MAMP by a *Nicotiana* LRR-RLP RXEG1 (Wang et al. 2018, Ma et al. 2015). There are a significant number of PRRs on the surface of plant cells and the ligands of most of these receptors remain unknown.

LRR-RLKs are also widespread in oomycetes, which include many plant pathogens belonging to genus *Phytophthora*, *Pythium*, and *Albugo* (Diévart et al. 2011; Si et al. 2021). For example, the *P. capsici* PcLRR-RK1 is required for hyphae growth, zoospore development, and full virulence of *P. capsici* (Safdar et al. 2017). In *P. sojae*, 15 out of 24 PsRLKs act in pathogenicity, zoospore development, and/or stress responses (Si et al. 2021). Surprisingly, Pei and colleagues (Pei et al. 2023) demonstrated that the *P. sojae* PsRLK6 functions as a MAMP to mediate cell-to-cell communications between the pathogen and the host.

Pei and colleagues expressed the extracellular domains (ECDs) of all 24 PsRLKs from *P. sojae* in *N. benthamiana* and found that PsRLK6^ECD^ and PsRLK7^ECD^ induced H_2_O_2_ accumulation in *N. benthamiana* leaves and enhanced resistance to *P. capsici* infection. However, when purified proteins of PsRLK6^ECD^ and PsRLK7^ECD^ were used for immune response analysis, only PsRLK6^ECD^ but not PsRLK7^ECD^ elicited burst of reactive oxygen species (ROS). Therefore, PsRLK6^ECD^ was selected for further investigation. Purified PsRLK6^ECD^ protein triggered other PTI (pattern-triggered immunity) responses in *N. benthamiana*, including phosphorylation of MAPK and upregulation of *PR1a* and *CYP71D20*, and consistently improved resistance to *P. capsici*. PsRLK6^ECD^-induced immune responses and *P. capsici* resistance were significantly compromised in *BAK1*-silenced or *sobir1* knockout *N. benthamiana* plants, indicating that *BAK1* and *SOBIR1* are required for PsRLK6^ECD^-triggered plant immunity. PsRLK6^ECD^ also induced ROS production, *PR1a* expression, and disease resistance in soybean and tomato, but not in Arabidopsis. To identify the critical region of PsRLK6 for eliciting plant immunity, Pei et al. used AlphaFold 2 to predict the 3D structure of PsRLK6^ECD^. Their data indicate that PsRLK6^ECD^ has a signal peptide domain, an LRR capping domain, seven atypical LRR domains, and an LRR C-terminal domain. By constructing a series of truncated mutant variants, Pei et al. identified that LRR5-6 is sufficient for inducing ROS accumulation and *P. capsici* resistance in *N. benthamiana*. Purified LRR5-6 peptide also induced ROS burst in soybean and tomato. Therefore, two LRR domains of PsRLK6^ECD^ are critical for its elicitor activity. Phylogenetic analysis showed that 53 PsRLK6 homologs were found in different oomycete species, but not in tested fungi, bacteria, and plants. The ECDs of PsRLK6 homologs from *Phytophthora infestans*, *Phytophthora capsici*, *Phytophthora parasitica*, *Phytophthora nicotianae*, *Phytophthora cactorum*, and *Pythium oligandrum* also induced H_2_O_2_ accumulation and *P. capsici* resistance in *N*. *benthamiana* leaves, suggesting that the elicitor activity of RLK6 is conserved in oomycetes.

To determine the role of PsRLK6 during *P. sojae*-soybean interaction, Pei et al. monitored the immune responses of soybean challenged with knockout mutant, overexpression, or wild-type *P. sojae* strains. Their data revealed that the overexpression strain induced higher ROS burst, *PR1a* expression, and MAPK activation than the wild-type and knockout mutant. In consistence, the virulence of the overexpression strain was severely compromised. However, the virulence of the knockout mutant was not affected compared to the wild-type. Then, what is the biological significance of PsRLK6 for *P. sojae*? As Pei et al. observed no obvious phenotypes of the knockout mutant *ΔPsRLK6* in filamentous growth and zoospore development, they further checked the role of PsRLK6 during the sexual reproduction of *P. sojae*. Pei et al. found that the production of oospores, which act as an important inoculum for the next growing season (Tyler 2007), was considerably reduced and morphologically impaired in the *ΔPsRLK6* mutant compared to the wild-type when cultured on lima bean agar medium or during infection with soybean. By contrast, oospore production was increased in the overexpression strains. Consistent with these results, the expression level of *PsRLK6* was significantly upregulated during oospore maturation on lima bean agar medium and in the late stages of infection on soybean roots. Altogether, PsRLK6 is required for oospore development in *P. sojae*.

**Fig. 1:**
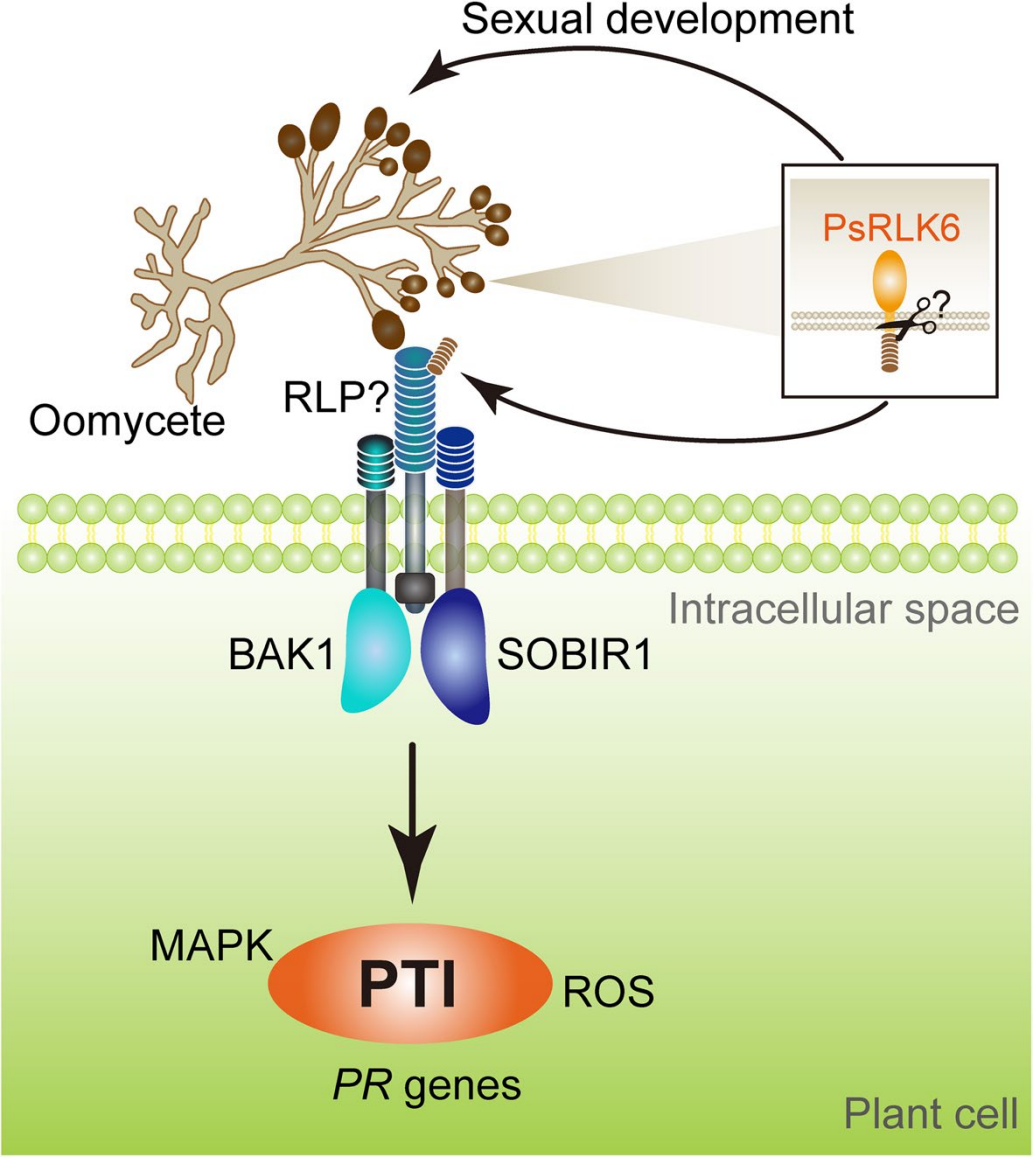
*P. sojae* cell membrane receptor is recognized by plant membrane receptors. *Phytophthora* cell membrane protein PsRLK6 may act as a sex hormone receptor to regulate the development of oospore. The PsRLK6 ECD may be released by an unknown mechanism and recognized by the plant cell membrane receptor kinase protein complex involving BAK1 and SOBIR1 to trigger PTI responses

In summary (Fig. 1), Pei et al. have demonstrated that an LRR-RLK PsRLK6 regulates the sexual development of *P. sojae* by enhancing the production of oospores, which is indicative of a reproductive or 'life' strategy as employed by plant RLKs during plant fertilization (Berger 2009). Additionally, PsRLK6 activates plant immunity pathways that rely on BAK1 and SOBIR1, leading to the suppression or death of *P. sojae*. The findings of this study are novel in that a receptor in pathogen is recognized by another receptor in plant, establishing a new type of MAMP that mediates the cell-to-cell communication between the pathogen and the plant. Unlike other MAMPs, which typically consist of secreted virulence factors or cell wall components (Yu et al. 2017), PsRLK6 is involved in a receptor-receptor interaction representing a unique mode signaling between the two organisms. However, it needs further investigation whether PsRLK6 carries protein kinase activity and acts as a genuine receptor kinase sensing signals that initiate sexual development of *P. sojae*. PsRLK6 is a transmembrane protein and its ECD may be cleaved and released into the plant apoplast where pathogen-plant communications might occur. The PsRLK6 ECD may be released by a controlled proteolysis process and sensed by a plant LRR-RLP, which deserve investigation in future studies. It would also be intriguing to investigate the mechanisms by which *P. sojae* coordinates PsRLK6-mediated sexual development and evasion of host immunity triggered by PsRLK6.

## Data Availability

Not applicable.

## Abbreviations

PTI: Pattern-triggered immunity
PRR: Pattern recognition receptor
MAMP: Microbe-associated molecular pattern
LRR-RLK: Leucine-rich repeat receptor-like kinase
LRR-RLP: Leucine-rich repeat receptor-like protein
ECD: Extracellular domain
ROS: Reactive oxygen species
